# Efficacy of Yijinjing-inspired exercises on sleep disorders in Parkinson’s disease: a controlled fNIRS study

**DOI:** 10.3389/fpsyt.2025.1470847

**Published:** 2025-07-18

**Authors:** Zekai Hu, Jing Shi, Jinyan Wang, Ting Liu, Yujia Li, Han Xue, Xueming Jin, Yiting Xue, Jie Wang, Xinhao Liu, Jun Hu

**Affiliations:** ^1^ Clinical Research Center, The Second Rehabilitation Hospital of Shanghai, Shanghai, China; ^2^ Department of Rehabilitation Medicine, Baoshan Branch, Renji Hospital affiliated with Shanghai Jiao Tong University School of Medicine, Shanghai, China; ^3^ Department of Rehabilitation Medicine, Baoshan Luodian Hospital of Shanghai, Shanghai, China; ^4^ School of Rehabilitation Science, Shanghai University of Traditional Chinese Medicine, Shanghai, China; ^5^ Pudong Medical Center, Pudong Hospital affiliated with Fudan University, Shanghai, China

**Keywords:** Parkinson's disease, sleep disorders, exercise, fNIRS, non-pharmacological intervention

## Abstract

**Background:**

Sleep disorders are prevalent in Parkinson's disease (PD) patients, significantly impacting their quality of life and rehabilitation outcomes. This controlled trial aimed to investigate the impact of Yijinjing-inspired exercises on sleep disorders in PD patients, utilizing functional near-infrared spectroscopy (fNIRS) to assess neurophysiological changes.

**Methods:**

Ninety-six PD patients were allocated to control, exercise, or music therapy groups for eight weeks. The Pittsburgh Sleep Quality Index (PSQI), along with the Montreal Cognitive Assessment (MoCA), Mini-Mental State Examination (MMSE), Unified Parkinson's Disease Rating Scale (UPDRS), and Parkinson's Disease Questionnaire (PDQ-39) were used to assess outcomes. fNIRS measured neurophysiological changes post-intervention.

**Results:**

The exercise group demonstrated substantial improvement in sleep quality after Bonferroni correction (PSQI: mean Δ = -1.78 ± 0.99, *P* < 0.001; Cohen's d = 1.45). Moderate effect sizes were observed in cognition (MoCA: d = 0.43) and motor function (UPDRS: d = 0.40), though these did not retain statistical significance after correction. Between-group analysis revealed greater PSQI reduction in exercise versus control (Δ = -1.19 ± 0.85 vs. -0.19 ± 1.53; *P* = 0.001 after Bonferroni adjustment, Cohen's d = 0.87), but not versus music therapy (*P* = 0.018 > 0.0167). fNIRS confirmed cortical reorganization in dorsolateral prefrontal cortex (Brodmann Area 9; Channel 3) and primary motor cortex (Brodmann Area 4; Channel 9) at FDR-corrected *P* < 0.05. The control group showed no statistically significant changes post-correction (all *P* > 0.01).

**Discussion:**

Yijinjing training may improve sleep quality in Parkinson's disease patients, with preliminary evidence of neuroplastic adaptation. Consideration could be given to exploring its integration into comprehensive rehabilitation approaches.

## 1 Introduction

Parkinson's disease (PD) is a progressive neurodegenerative disorder affecting millions globally (1), presenting a range of motor and non-motor symptoms (2). Among these, sleep disorders are notably prevalent, with epidemiological studies suggesting that over 60% of PD patients experience varying degrees of sleep-related problems (3). Such disturbances can stem from multiple factors: the degeneration of specific brain regions responsible for sleep regulation (4), side effects from medication (5), and secondary consequences of motor symptoms, such as nocturnal akinesia (6).

The consequences of sleep disorders in PD patients are profound, often exacerbating motor and cognitive symptoms (7), thereby significantly deteriorating their quality of life (8). Moreover, inadequate sleep has been associated with decreased functional capacity and poorer rehabilitation outcomes in these individuals (9). Hence, addressing sleep disorders is crucial not only for the patients' well-being but also for optimizing their rehabilitation potential.

Traditionally, rehabilitation interventions for PD have focused primarily on motor symptoms. However, while pharmacological agents remain a mainstay for managing sleep disturbances, their long-term efficacy is often limited by side effects such as dependency and daytime somnolence (10). Recent literature has emphasized the value of holistic approaches, considering both motor and non-motor symptoms, with emerging evidence suggesting that non-pharmacological interventions like integrative mind-body exercises and music therapy may uniquely target neural circuits involved in sleep regulation (11). Among these interventions, certain exercise-based approaches, which are similar to the principles of 'Yijinjing' exercises—a form of physical exercise known for its coordination and flexibility-enhancing attributes—have been proposed as potential strategies to address sleep disorders (12). Music therapy, on the other hand, has been recognized for its potential in regulating mood and improving sleep quality, due to its influence on the brain's neurochemical processes (13).

Yijinjing, originating from ancient Chinese martial traditions documented in the Ming Dynasty text The Illustrated Exposition of Internal Techniques, emphasizes dynamic tendon-strengthening through isometric contractions and controlled breathing rhythms (14). Unlike Tai Chi's continuous flowing movements focused on balance, Yijinjing employs sustained postural holds (e.g., 'Pulling Nine Oxen Tails') to enhance proprioceptive feedback and corticospinal facilitation (15). This sustained biomechanical loading during postural holds induces fascial tension that propagates along myofascial chains, potentially transmitting mechanical stress through craniocervical interfaces to modulate meningeal mechanotransduction. As evidenced by intracranial recordings, such mechanosensitive signaling can enhance thalamocortical arousal-regulating oscillations while dampening hyperexcitability in rigidity-associated sensorimotor cortices (16). This specific neuromuscular modulation may uniquely benefit PD patients with rigidity-dominant phenotypes.

While the aforementioned therapeutic interventions have been previously explored, the underlying neurophysiological changes in the brain remain to be fully understood (17). The Functional Alterations in Low-Frequency Fluctuations (fALFF), as derived from resting-state functional near-infrared spectroscopy, provides a novel avenue to probe these mechanisms at the cortical level (18). It offers an opportunity to directly observe the changes in brain functionality after interventions, potentially linking therapy outcomes with neurophysiological adaptions (19).

We hypothesize that Yijinjing-inspired exercises will result in significant improvements in sleep quality, motor function, and cognitive function in PD patients, and that these improvements will be associated with measurable changes in neurophysiological activity as assessed by fNIRS. In light of this, this controlled trial aimed to investigate the impact of Yijinjing-inspired exercises on sleep disorders in PD patients, utilizing fNIRS to assess neurophysiological changes.

## 2 Materials and methods

### 2.1 Study population and design

We conducted our study with 96 Parkinson's disease (PD) patients admitted to the Baoshan Branch of Ruijin Hospital affiliated to the Shanghai Jiao Tong University School of Medicine between December 2020 and June 2024. The inclusion criteria were: (1) Diagnosis based on the Chinese Parkinson's Disease Diagnostic Criteria (2016 edition); (2) Availability of complete clinical data; (3) Compliance with the Helsinki Declaration for ethical review standards; (4) Pittsburgh Sleep Quality Index (PSQI) score > 7; and (5) Absence of significant heart, liver, or kidney function abnormalities.

Exclusion criteria were: (1) Patients with serious organic lesions in the heart, liver, or kidneys; (2) Patients with speech or consciousness disorders; (3) Patients currently participating in other experimental intervention studies that could potentially influence the outcomes of this study; and (4) Patients with sleep disorders attributed to other diseases, surgeries, tumors, or external factors.

All groups, including the control, exercise, and music therapy groups, received standard conventional rehabilitation therapy at our hospital, which includes physical therapy (PT), occupational therapy (OT), and speech therapy (ST), to maintain ethical standards and control variables. This ensures that all participants received a consistent level of baseline care while the effects of the additional interventions were evaluated.

### 2.2 Ethical statement

This study was reviewed and approved by the Ethical Committee of The Second Rehabilitation Hospital of Shanghai(No.2020-0501) and the Ethics Committee of the Baoshan Branch of the Renji Hospital affiliated with the Shanghai Jiao Tong University School of Medicine (No.2020-qkwkt-005). All participants signed an informed consent form.

### 2.3 Sample size

Sample size determination was performed using G*Power 3.1 software for *a priori* power analysis. We specified a medium effect size (Cohen's d = 0.5) based on established methodological standards in Parkinson's rehabilitation research, where this magnitude corresponds to clinically meaningful thresholds for sleep outcomes. The analysis parameters (α = 0.05, power = 0.80, three groups, one primary endpoint PSQI) yielded a requirement of 84 participants. To accommodate a conservative 15% attrition rate aligned with PD exercise trial averages, we recruited 96 participants with equal allocation to control, exercise, and music therapy groups (n = 32 per group).

### 2.4 Randomization and blinding

Eligible participants were randomly assigned to one of the three groups (control, exercise, music therapy) using a computer-generated randomization sequence with block size of 6, prepared and concealed by an independent researcher not involved in recruitment or assessment. Outcome assessors responsible for administering the PSQI, MoCA, MMSE, UPDRS, and PDQ-39 scales were blinded to group allocation. Due to the distinct nature of the interventions, participants and therapists delivering the interventions were not blinded. The fNIRS data acquisition followed a standardized protocol, and the subsequent fALFF analysis was performed using the NIRS_KIT software with predefined parameters by an analyst blinded to group assignment.

### 2.5 Demographic distribution and baseline characteristics

The 96 participants were systematically divided into three groups: control, exercise (emulating 'Yijinjing' principles), and music therapy, each comprising 32 individuals. The control group consisted of 19 males and 13 females with an average age of 69.87 years (SD = 3.42). The exercise group was composed of 18 males and 14 females, with an average age of 67.59years (SD = 4.94). The music therapy group included 18 males and 14 females with an average age of 69.40 years (SD = 5.47). A one-way ANOVA analysis revealed no significant differences in age distribution among the groups (*P* = 0.127, F = 2.108).

Furthermore, one-way ANOVA analysis showed no significant differences in the baseline scores across various scales among the control, exercise, and music therapy groups. Specifically, there were no significant differences in the MoCA scale scores (F=0.062, *P* = 0.940), MMSE scale scores (F=0.098, *P* = 0.906), PSQI scale scores (F=0.449, *P* = 0.640), UPDRS scale scores (F=0.978, *P* = 0.380), and PDQ-39 scale scores (F=0.635, *P* = 0.532). These results indicate that the three groups were similar in their cognitive levels, Parkinson's disease stages, and the severity of sleep disorders at baseline, with no statistically significant differences.

### 2.6 Intervention procedures

All the study participants underwent routine drug treatment and maintained their regular daily life schedules.

#### 2.6.1 Control group

The control group received conventional rehabilitation training comprising: physical therapy (10 min: balance training including semi-tandem stance and weight shifting; gait training with obstacle negotiation and visual cueing), occupational therapy (10 min: activities of daily living retraining such as buttoning and utensil use; fine motor coordination using nine-hole pegboard), and speech therapy (10 min: respiratory-phonatory coordination through sustained vowel phonation; orofacial exercises). This was supplemented with 30 minutes of structured insomnia health education covering sleep hygiene principles, stimulus control techniques, and relaxation methods. The total 60-minute intervention was administered once daily, five days a week, for eight weeks.

#### 2.6.2 Exercise group

**Figure 1:**
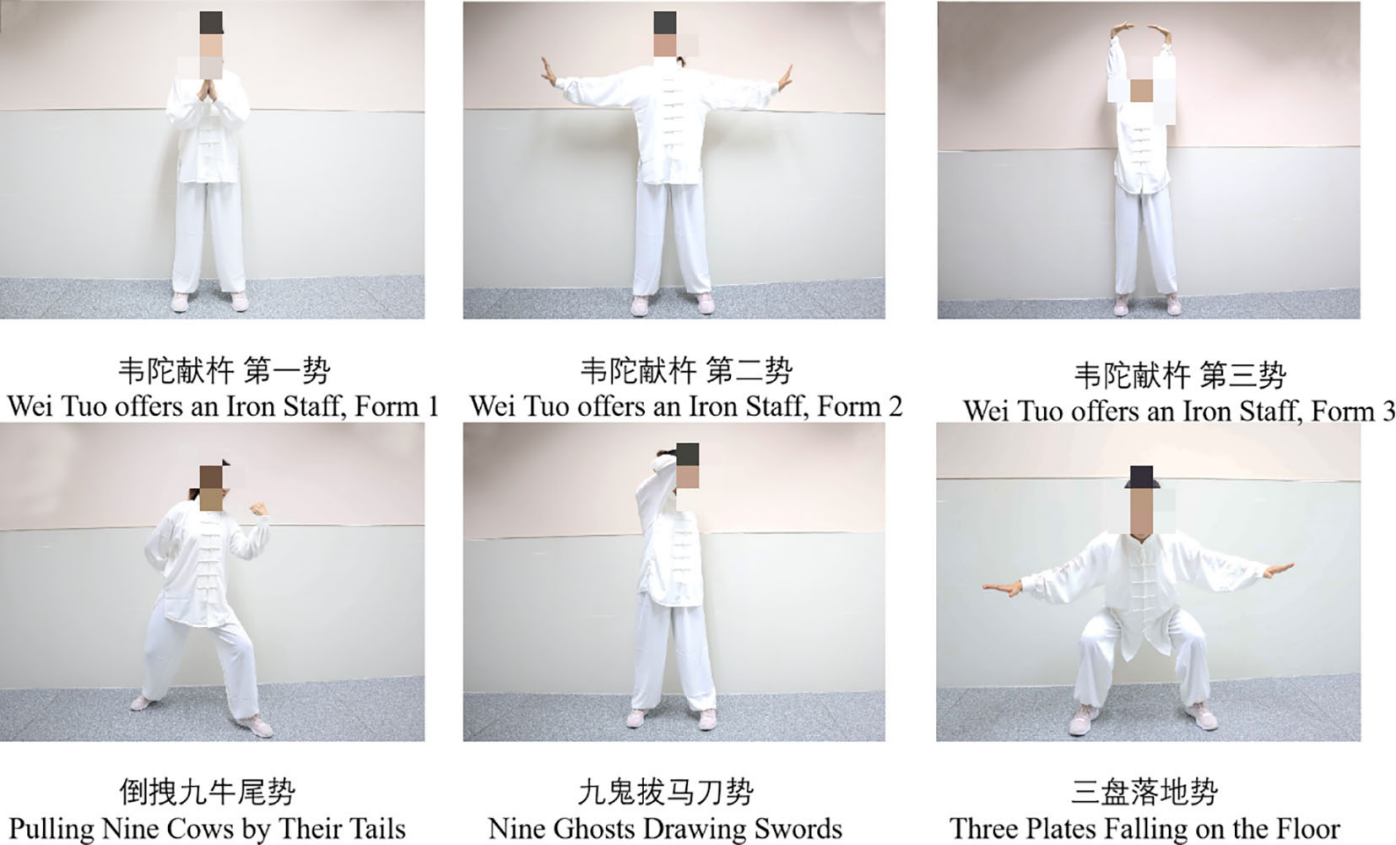
Schematic diagram of Yijinjing-inspired exercises.

In addition to the conventional rehabilitation training, this group incorporated physical exercises inspired by the traditional Yijinjing practices. Our team selected six biomechanically-standardized motions suitable for PD patients: (1) Vertical Hand Elevation: Starting in neutral stance, hands elevated vertically at 15°/sec to 120° shoulder flexion with scapular control, maintaining 10° knee flexion (3 repetitions × 15-second holds); (2) Lateral Tension Stretch: Bilateral arm abduction to 90° at 30° external rotation, synchronized with diaphragmatic breathing (4-second inhalation:6-second exhalation ratio) during 5 repetitions; (3) Overhead Extension: Full scapulohumeral rhythm with bilateral elevation to 170°flexion, maintaining lumbo-pelvic stability through 20° hip hinge (5 repetitions × 8-second cycles); (4) Step-Pull Sequence: Half-step forward at controlled cadence (30 cm step distance), performing isometric scapular retraction at 70% maximum voluntary contraction during claw-grip sustain (thumb-index opposition at 20N force); (5) Axial Rotation: Controlled 45° thoracic rotation with contra-lateral hand reaching mastoid level, maintaining pelvic stability (3 sets × 5 rotations/side); (6) Centered Squat: Squat descent to 25° knee flexion with eccentric control (5-sec descent), arms extended anteriorly at 60° elevation. Each 30-minute session followed standardized cadence: 5-min warm-up → 20-min movement execution → 5-min recovery. The training was provided once daily, five days a week, for eight consecutive weeks. See **Figure 1**.

#### 2.6.3 Music group

Drawing on the findings of previous studies, our music therapy protocol was derived from the principles outlined by Särkämö et al. (2014) (20). Patients participated in standardized music therapy sessions implementing evidence-based neuroacoustic protocols calibrated for PD populations. The auditory stimuli comprised three distinct categories with strict selection criteria: classical compositions (70% session duration, e.g., Debussy's Clair de Lune), jazz standards (20% duration, limited to cool jazz subgenre like Miles Davis' Blue in Green), and Chinese folk melodies (10% duration, e.g., pentatonic-scale Jasmine Folk Song). All pieces underwent technical validation to ensure adherence to 60-80 BPM. Sessions were delivered in acoustically controlled environments (background noise ≤30dB) using noise-cancelling headphones, with continuous 30-minute playback at 60 dB volume without therapist interruption. This standardized protocol was administered once daily, five days per week, for eight consecutive weeks.

### 2.7 Intervention fidelity monitoring

To ensure the interventions were delivered as intended and to assess participant adherence, the following fidelity measures were implemented:

Attendance: Participant attendance at every session was meticulously recorded using a standardized attendance log. Adherence was calculated as the percentage of scheduled sessions attended.

Therapist Training and Protocol Adherence: All therapists underwent standardized training based on detailed intervention manuals. These manuals specified the exact sequence, duration, key components (e.g., specific Yijinjing-inspired movements, music selection criteria, insomnia education topics), and instructions for each session. To monitor delivery fidelity, the research coordinator conducted periodic, unannounced observational visits (at least one session per therapist per month) using a structured fidelity checklist. Additionally, therapists completed a brief session log after each intervention documenting completion of core components and any deviations or participant issues.

Participant Compliance: During sessions, therapists ensured participants engaged appropriately (performing exercises to the best of their ability, listening attentively during music sessions). For the exercise group, therapists provided corrections if movement execution deviated significantly from the standard.

### 2.8 Safety monitoring

Adverse events were systematically documented using a standardized protocol aligned with CONSORT guidelines. Therapists recorded any intervention-related incidents (falls, musculoskeletal pain and dizziness) during daily sessions. Events were categorized by severity (mild: self-resolving; moderate: requiring modification; severe: discontinuation) and causality (related/unrelated to intervention). All participants received safety education on movement modifications and fall prevention strategies prior to intervention commencement.

### 2.9 Assessment techniques

#### 2.9.1 Scale evaluation

Several scales were utilized to assess the participants:

MoCA: The Montreal Cognitive Assessment, a brief 30-question test that assesses different cognitive domains.

MMSE: The Mini-Mental State Examination, commonly used to screen for cognitive impairment.

PSQI: Pittsburgh Sleep Quality Index, a tool to measure the quality and patterns of sleep.

UPDRS: Unified Parkinson’s Disease Rating Scale, which measures both motor and non-motor symptoms of PD.

PDQ-39: Parkinson's Disease Questionnaire, a 39-item instrument that assesses the health status of PD patients2.

#### 2.9.2 fNIRS imaging

##### 2.9.2.1 Data acquisition

A 22-channel portable fNIRS imaging device (lightnirs, Shimadzu) was used. It incorporated eight light-emitting and eight detecting probes, forming 22 effective channels, spaced 30mm apart, primarily covering the frontal lobe. The central brain area beneath the midpoint of the connecting line between the emitter and detector was considered the main detection zone, labeled according to the Brodmann cerebral cortical areas. During scanning, subjects sat in a relaxed state with closed eyes, ensuring minimal cognitive activity. Baseline resting-state data were collected for 8 minutes before and after task exercises.

##### 2.9.2.2 Data analysis

Collected fNIRS raw data were processed using the NIRS_KIT software (21) on Matlab 2018b^3^. Procedures involved integrating raw fNIRS signal time-series, hemoglobin concentration changes, and spatial geometry. Drifts were removed using polynomial regression models, and motion artifacts were corrected with temporal derivative distribution repair (TDDR). Motion artifact correction implemented a 3.0 standard deviation threshold for spike removal. Hemoglobin concentration changes were derived using the modified Beer-Lambert law with differential pathlength factors set at 6.0 (780 nm) and 5.2 (850 nm). Baseline normalization utilized whole-session mean scaling prior to fALFF computation.The signal intensity of oxygenated hemoglobin at the individual level was analyzed by executing fALFF, setting the low-frequency fluctuation amplitude between 0.01 to 0.08Hz.The 0.01-0.08 Hz frequency band for fALFF analysis was selected based on established protocols for PD neuroimaging studies, as this range optimally captures slow cortical hemodynamic oscillations associated with neuroplastic changes while minimizing contamination from higher-frequency physiological noise (cardiac cycles > 0.1 Hz, respiration 0.1-0.3 Hz) (22). This bandwidth has demonstrated particular sensitivity to cortico-striatal connectivity alterations in PD populations during resting-state assessments.

### 2.10 Statistical analysis

Data were analyzed using SPSS 22.0 software (IBM Corp., Armonk, NY). Baseline characteristics across groups were evaluated using one-way analysis of variance (ANOVA). For within-group comparisons, paired-sample t-tests assessed pre-post intervention changes in clinical scales (MoCA, MMSE, PSQI, UPDRS-III, PDQ-39), with Bonferroni correction applied to control Type I error inflation from multiple comparisons; the significance threshold was adjusted to α = 0.01 per group (0.05/5 scales). Effect sizes for significant within-group differences were calculated using Cohen’s d, where d = mean change score / standard deviation of change scores. Between-group differences were analyzed using one-way ANOVA, with effect sizes quantified by partial η^2^ When ANOVA indicated significant differences (*P* < 0.05), Bonferroni-adjusted *post hoc* tests were conducted for pairwise comparisons, with the significance threshold set at α = 0.0167 per scale (0.05/3 pairwise tests). Cohen’s d was additionally computed for significant between-group differences using pooled standard deviations. For fNIRS data, false discovery rate (FDR) correction was applied during fALFF analysis in NIRS_KIT, while all other analyses used two-tailed tests with α = 0.05. Interpretation of effect sizes followed established conventions: Cohen’s d thresholds were 0.2 (small), 0.5 (medium), and 0.8 (large); partial η^2^ thresholds were 0.01 (small), 0.06 (medium), and 0.14 (large).

## 3 Results

### 3.1 Intervention fidelity and compliance

Participant adherence to the intervention schedule was high. The mean attendance rates were 92.4% (SD = 5.1%) for the exercise group, 94.1% (SD = 4.3%) for the music group, and 90.7% (SD = 6.8%) for the control group, with no significant between-group differences (F=2.15, *P* = 0.122). Three participants withdrew (one from each group) due to personal reasons unrelated to the study.

Observations (n=48 sessions) and therapist logs indicated excellent protocol adherence. Minor deviations (e.g., session shortened by <5 minutes due to participant fatigue on rare occasions, slight individual adaptation of an exercise) were infrequent and documented. Structured fidelity checks during observational visits confirmed that >95% of core intervention components were consistently delivered across all sessions and therapists. No severe adverse events occurred across all groups. Three mild events were documented in the exercise group (two cases of transient muscle soreness, one incident of mild dizziness resolving within 10 minutes), representing 3.1% of exercise participants. No modifications or discontinuations were required. The music and control groups reported zero adverse events.

### 3.2 Within-group comparison

In the exercise group, significant improvements were observed across multiple domains: MoCA scores increased from 20.34 ± 1.64 to 20.78 ± 1.56 (mean difference 0.44, 95% CI 0.07-0.81; t = -2.44, *P* = 0.021), MMSE scores from 21.22 ± 1.90 to 21.69 ± 1.87 (difference 0.47, 95% CI 0.11-0.83; t = -2.61, *P* = 0.014), PSQI scores decreased from 16.81 ± 1.26 to 15.03 ± 1.23 (difference -1.78, 95% CI -2.06 to -1.50; t = 11.58, *P* < 0.001), UPDRS scores from 16.72 ± 1.75 to 15.84 ± 1.87 (difference -0.88, 95% CI -1.67 to -0.09; t = 2.24, *P* = 0.032), and PDQ-39 scores from 34.91 ± 3.48 to 33.59 ± 3.43 (difference -1.32, 95% CI -2.48 to -0.16; t = 2.30, *P* = 0.028). The music group demonstrated a modest reduction in PSQI scores from 16.84 ± 1.30 to 16.31 ± 1.35 (difference -0.53, 95% CI -1.01 to -0.05; t = 2.28, *P* = 0.031) but showed no significant changes in other measures. No statistically significant within-group changes were detected in the control group across all assessments. See **Table 1**.

**Table 1:** Results of inter-group scale statistical comparison.

Group	Scale	Pre-treatment	Post-treatment	T	*P*	Cohen's d
Control Group	MoCA, Mean ± SD	20.66 ± 1.79	20.91 ± 1.82	-0.80	0.428	0.142
	MMSE, Mean ± SD	21.03 ± 1.86	21.25 ± 1.81	-1.31	0.198	0.232
	PSQI, Mean ± SD	16.53 ± 2.08	16.34 ± 2.17	0.69	0.494	0.122
	UPDRS, Mean ± SD	17.09 ± 1.53	17.03 ± 1.80	0.22	0.827	0.039
	PDQ-39, Mean ± SD	33.94 ± 3.90	33.56 ± 4.49	0.39	0.701	0.068
Exercise Group	MoCA, Mean ± SD	20.34 ± 1.64	20.78 ± 1.56	-2.44	0.021	0.431
	MMSE, Mean ± SD	21.22 ± 1.90	21.69 ± 1.87	-2.61	0.014	0.462
	PSQI, Mean ± SD	16.81 ± 1.26	15.03 ± 1.23	11.58	<.001^†^	1.447
	UPDRS, Mean ± SD	16.72 ± 1.75	15.84 ± 1.87	2.24	0.032	0.396
	PDQ-39, Mean ± SD	34.91 ± 3.48	33.59 ± 3.43	2.30	0.028	0.407
Music Group	MoCA, Mean ± SD	20.50 ± 1.63	20.78 ± 1.95	-1.39	0.174	0.246
	MMSE, Mean ± SD	21.19 ± 1.67	21.53 ± 1.52	-1.51	0.140	0.267
	PSQI, Mean ± SD	16.84 ± 1.30	16.31 ± 1.35	2.28	0.031	0.403
	UPDRS, Mean ± SD	17.28 ± 1.63	17.09 ± 1.94	0.51	0.617	0.089
	PDQ-39, Mean ± SD	34.22 ± 3.20	33.91 ± 3.35	0.45	0.653	0.080

T, t-test.

SD, standard deviation.

^†^Bonferroni-adjusted significance: within-group *P* ≤ 0.01.

Effect size interpretation: 0.2 (small), 0.5 (medium), 0.8 (large).

### 3.2 Between-group comparisons

Analysis of covariance revealed no between-group differences in changes for MoCA (F = 0.18, *P* = 0.837), MMSE (F = 0.42, *P* = 0.658), UPDRS (F = 1.55, *P* = 0.218), or PDQ-39 scores (F = 0.54, *P* = 0.584). However, significant between-group differences emerged for PSQI score changes (F = 5.20, *P* = 0.007), with partial η² = 0.10 indicating a medium-to-large effect. *Post-hoc* comparisons demonstrated a large effect size between exercise and control groups (mean difference -1.01, 95% CI -1.57 to -0.43; t = 3.47, *P* = 0.001; Cohen's d = 0.87), while exercise versus music comparison showed a medium effect (mean difference -0.66, 95% CI -1.20 to -0.12; t = -2.43, *P* = 0.018; Cohen's d = -0.53). Control versus music comparison was non-significant (*P* = 0.198). See **Tables 2** and **3**.

**Table 2:** Results of inter-group scale statistical comparison.

Variables	Total (n = 96)	Control group (n = 32)	Exercise group (n = 32)	Music group (n = 32)	F	*P*	Partial η^2^
ΔMoCa, Mean ± SD	0.32 ± 1.33	0.25 ± 1.76	0.44 ± 1.01	0.28 ± 1.14	0.18	0.837	0.004
ΔMMSE, Mean ± SD	0.34 ± 1.08	0.22 ± 0.94	0.47 ± 1.02	0.34 ± 1.29	0.42	0.658	0.009
ΔPSQI, Mean ± SD	-0.64 ± 1.31	-0.19 ± 1.53	-1.19 ± 0.82	-0.53 ± 1.32	5.20	0.007^†^	0.101
ΔUPDRS, Mean ± SD	-0.38 ± 2.00	-0.06 ± 1.61	-0.88 ± 2.21	-0.19 ± 2.10	1.55	0.218	0.032
ΔPDQ-39, Mean ± SD	-0.67 ± 4.29	-0.38 ± 5.48	-1.31 ± 3.23	-0.31 ± 3.90	0.54	0.584	0.012

F: ANOVA.

SD, standard deviation.

^†^Bonferroni-adjusted significance:between-group *P* ≤ 0.0167.

Partial η^2^ interpretation: 0.01 (small), 0.06 (medium), 0.14 (large).

**Table 3:** *Post-hoc* analysis of ΔPSQI between groups with effect sizes.

Comparison	Mean difference (95% CI)	T	*P*	Cohen’s d	Significance (α = 0.0167)
Control vs. Exercise​	1.01 (0.43, 1.57)	3.47	0.001	0.87	Significant
Control vs. Music​​	0.34 (-0.18, 0.86)	1.30	0.198	0.29	Non-significant
Exercise vs. Music​	-0.66 (-1.20, -0.12)	-2.43	0.018	-0.53	Marginal

T, t-test.

### 3.3 Functional near-infrared spectroscopy analysis

**Figure 2:**
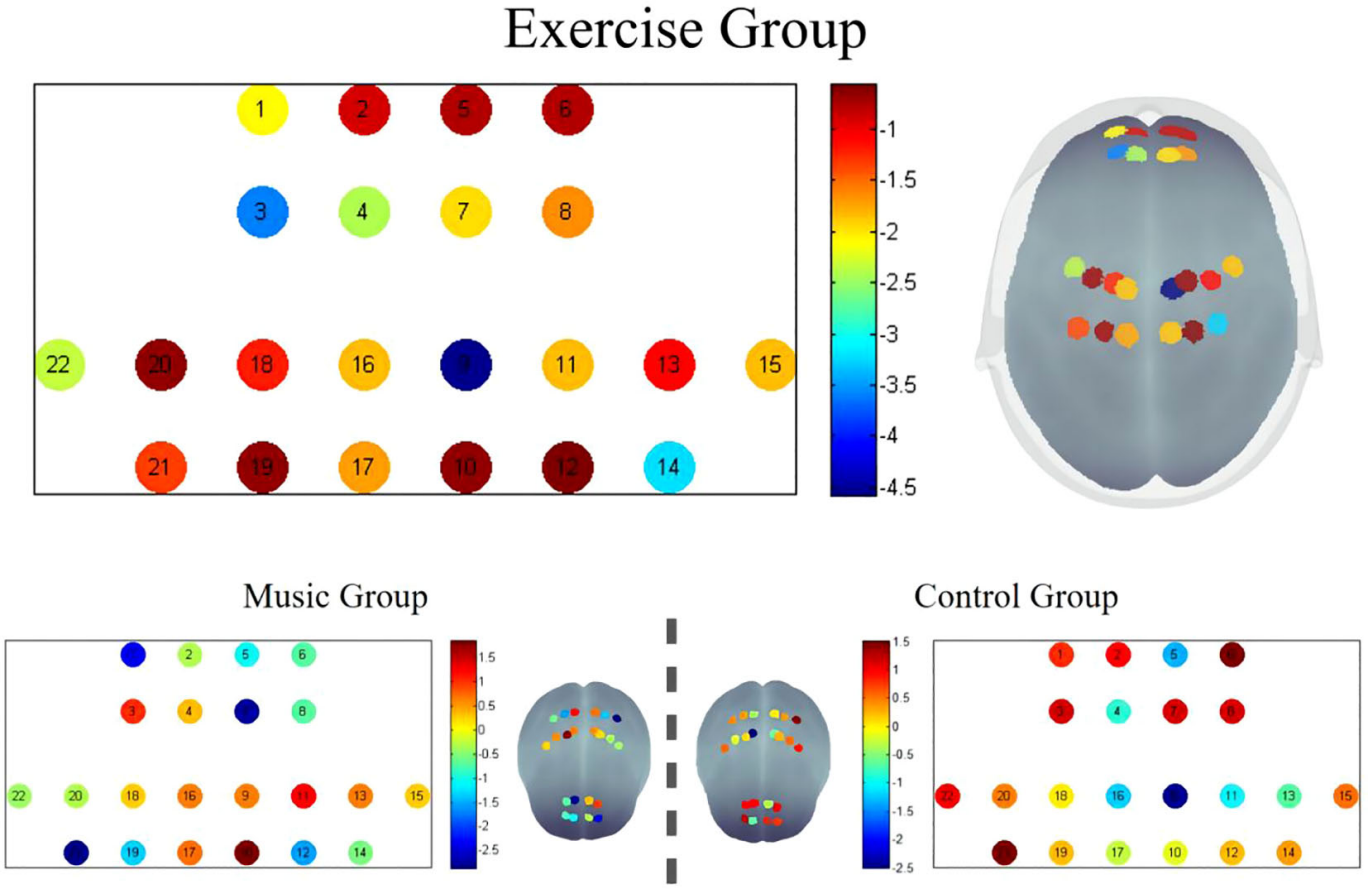
Changes in the fALFF before and after treatment.

After False Discovery Rate (FDR) multiple comparison correction, significant post-intervention differences were identified in the Exercise Group, particularly in channels 3, 9, and 14. The Montreal Neurological Institute (MNI) coordinates for these channels were channel 3 (-15; 54; 42,BA9), channel 9 (12; -16; 78,BA4), and channel 14 (34; -33; 73,BA7). These channels showed marked post-intervention improvements, with the remaining channels also exhibiting enhanced values compared to pre-intervention levels. The graphical representation of the fALFF values visually corroborates these findings, demonstrating localized variations in brain activity.In contrast, there were no significant differences in the fALFF values post-intervention in the control and music groups. See **Figure 2**.

## 4 Discussion

The current findings demonstrate that Yijinjing-inspired exercises significantly improved sleep quality in Parkinson's disease (PD) patients, aligning with existing evidence supporting mind-body interventions in PD rehabilitation (23). Specifically, integrative practices like Yijinjing that emphasize coordinated movement and mindfulness may enhance neuroplasticity through multiple pathways. Similar mechanisms have been observed in Tai Chi, where rhythmic physical activity can stabilize circadian rhythms (24). While our data also showed improvements in cognitive and motor functions, these did not retain statistical significance after rigorous correction for multiple comparisons, suggesting that sleep quality may represent the most responsive domain to this intervention within the current study parameters.

Neurophysiological adaptations observed via functional near-infrared spectroscopy provide correlative evidence that may help elucidate these clinical outcomes. Elevated fALFF values in the exercise group, particularly in the prefrontal cortex (channel 3) and supplementary motor area (channel 9), were associated with clinical improvements. These regions are critically involved in sleep regulation and motor control (25), and their activation patterns align with neuroimaging studies demonstrating altered functional connectivity in sensorimotor circuits following mind-body training (26). For instance, prior research on Tai Chi has shown optimization of intrinsic brain architecture, particularly in frontal regions (27). Notably, while Tai Chi primarily enhances functional connectivity within frontal networks, our observed activation in primary motor cortex (Brodmann Area 4) suggests Yijinjing's emphasis on sustained postural loading may uniquely engage corticospinal facilitation circuits. This divergence implies that different mind-body practices recruit complementary neuroplastic pathways—with Tai Chi optimizing network integration and Yijinjing strengthening elemental motor control. Such differential targeting could inform personalized rehabilitation approaches based on phenotypic profiles.However, it must be emphasized that these neurophysiological changes demonstrate correlation rather than established causation with functional improvements, as the temporal resolution of our measurements cannot confirm directional relationships between neural adaptation and behavioral outcomes.

The large effect size observed in PSQI improvements exceeds established thresholds for clinical significance in sleep interventions. Notably, this effect magnitude surpasses the minimum clinically important difference for PSQI in PD populations, suggesting meaningful sleep quality enhancement beyond statistical significance. The significantly greater reduction in PSQI scores compared to both control conditions—including an active control receiving insomnia education—underscores the potentially robust effect of the Yijinjing-based intervention.

In contrast, music therapy demonstrated more limited efficacy in this trial. The observed modest reduction in PSQI scores without significant effects on other measures suggests that while auditory stimulation may provide some benefit, its mechanisms likely differ from those engaged by motor-cognitive interventions (28). This discrepancy highlights the potential value of therapies that simultaneously target multiple neural circuits, though the precise factors underlying differential treatment response require further investigation. We acknowledge that our original explanation regarding neural oscillation misalignment represented speculative interpretation beyond direct evidence, and have reframed this conclusion accordingly.

The control group's lack of significant improvement underscores the limitations of conventional rehabilitation approaches for addressing sleep pathophysiology in PD. Traditional interventions often prioritize motor symptoms over holistic neuromodulation (29), whereas Yijinjing's integration of physical and cognitive engagement may address multifactorial sleep dysregulation more effectively (30). This aligns with increasing calls for integrative strategies in neurodegenerative disease management.

Several limitations warrant consideration when interpreting these findings. First, the 8-week intervention period may be insufficient to evaluate long-term neuroplasticity or sustained benefits (31). Second, the lack of participant and interventionist blinding introduces possible performance bias, though we mitigated assessment bias through blinded outcome evaluation. Third, fNIRS coverage was limited to frontal regions, potentially missing relevant network-level changes in other brain areas. Fourth, while we maintained stable pharmacologic regimens across all participants throughout the study, we cannot exclude potential interactions between dopaminergic medications and exercise interventions that might influence outcomes. Future studies with medication stratification could clarify this relationship. Finally, the homogeneous sample of Chinese patients necessitates cross-cultural validation of these findings.

Regarding our control group design, the additional insomnia health education sessions provided to this group represent a potential confounding factor that may have attenuated between-group differences. While this element aimed to reflect standard clinical practice for PD patients with sleep concerns, future research would benefit from implementing attention-controlled conditions that more rigorously isolate specific intervention effects.

Despite these limitations, this study provides evidence that Yijinjing-inspired training may meaningfully improve sleep quality in PD patients. The correlative neurophysiological data suggest possible neural mechanisms worthy of further investigation. These findings support continued exploration of non-pharmacological, multimodal approaches to PD management, with particular attention to sleep-related outcomes that significantly impact quality of life. Future longitudinal studies with broader neuroimaging coverage and medication monitoring may help establish causal mechanisms and optimize intervention protocols.

## 5 Conclusion

By integrating clinical and neurophysiological data, this study underscores Yijinjing’s role in ameliorating PD-related sleep disorders. The observed neural and functional improvements advocate for non-pharmacological, multimodal therapies in PD care, resonating with broader efforts to address neurodegenerative complexity.

## Data Availability

The original contributions presented in the study are included in the article/**Supplementary Material**. Further inquiries can be directed to the corresponding authors.

## References

[1] de Lau LM Breteler MM. Epidemiology of Parkinson's disease. Lancet Neurol. (2006) 5:525–35. doi: 10.1016/S1474-4422(06)70471-9 16713924

[2] Dorsey ER Bloem BR. The parkinson pandemic-A call to action. JAMA Neurol. (2018) 75:9–10. doi: 10.1001/jamaneurol.2017.3299 29131880

[3] Chahine LM Amara AW Videnovic A. A systematic review of the literature on disorders of sleep and wakefulness in Parkinson's disease from 2005 to 2015. Sleep Med Rev. (2017) 35:33–50. doi: 10.1016/j.smrv.2016.08.001 27863901 PMC5332351

[4] Schrempf W Brandt MD Storch A Reichmann H. Sleep disorders in Parkinson's disease. J Parkinsons Dis. (2014) 4:211–21. doi: 10.3233/JPD-130301 24796235

[5] Li BD Bi ZY Liu JF Si WJ Shi QQ Xue LP. Adverse effects produced by different drugs used in the treatment of Parkinson's disease: A mixed treatment comparison. CNS Neurosci Ther. (2017) 23:827–42. doi: 10.1111/cns.12727 PMC6492757 28872217

[6] Sveinbjornsdottir S. The clinical symptoms of Parkinson's disease. J Neurochemistry. (2016) 139:318–24. doi: 10.1111/jnc.13691 27401947

[7] Kurtis MM Rodriguez-Blazquez C Martinez-Martin P. Relationship between sleep disorders and other non-motor symptoms in Parkinson's disease. Parkinsonism Relat Disord. (2013) 19:1152–5. doi: 10.1016/j.parkreldis.2013.07.026 23953775

[8] Kay DB Tanner JJ Bowers D. Sleep disturbances and depression severity in patients with Parkinson's disease. Brain Behav. (2018) 8:e00967. doi: 10.1002/brb3.967 30106239 PMC5991567

[9] Abbruzzese G Marchese R Avanzino L Pelosin E. Rehabilitation for Parkinson's disease: Current outlook and future challenges. Parkinsonism Relat Disord. (2016) 22 Suppl 1:S60–4. doi: 10.1016/j.parkreldis.2015.09.005 26360239

[10] Goodwin VA Richards SH Taylor RS Taylor AH Campbell JL. The effectiveness of exercise interventions for people with Parkinson's disease: a systematic review and meta-analysis. Mov Disord. (2008) 23:631–40. doi: 10.1002/mds.21922 18181210

[11] Snider J Müller ML Kotagal V Koeppe RA Scott PJ Frey KA. Non-exercise physical activity attenuates motor symptoms in Parkinson disease independent from nigrostriatal degeneration. Parkinsonism Relat Disord. (2015) 21:1227–31. doi: 10.1016/j.parkreldis.2015.08.027 PMC4587298 26330028

[12] Chen S Zhang Y Wang YT Liu X Song W Du X. The effect of Qigong-based therapy on patients with Parkinson's disease: a systematic review and meta-analysis. Clin Rehabil. (2020) 34:1436–48. doi: 10.1177/0269215520946695 32727214

[13] Thaut MH McIntosh GC Hoemberg V. Neurobiological foundations of neurologic music therapy: rhythmic entrainment and the motor system. Front Psychol. (2014) 5:1185. doi: 10.3389/fpsyg.2014.01185 25774137 PMC4344110

[14] Li L Liang J Fan T. Effect of five traditional Chinese medicine exercises on insomnia: A systematic review and network meta-analysis. J Psychiatr Res. (2025) 181:312–9. doi: 10.1016/j.jpsychires.2024.12.004 39642468

[15] Jiang B Feng C Hu H George D Huang T Li Z. Traditional chinese exercise for neurodegenerative diseases: A bibliometric and visualized analysis with future directions. Front Aging Neurosci. (2022) 14:932924. doi: 10.3389/fnagi.2022.932924 35832067 PMC9271864

[16] He J Chan SH Lin J Tsang HW. Integration of tai chi and repetitive transcranial magnetic stimulation for sleep disturbances in older adults: A pilot randomized controlled trial. Sleep Med. (2024) 122:35–44. doi: 10.1016/j.sleep.2024.07.029 39121822

[17] McGregor MM Nelson AB. Circuit mechanisms of Parkinson's disease. Neuron. (2019) 101:1042–56. doi: 10.1016/j.neuron.2019.03.004 30897356

[18] Maidan I Nieuwhof F Bernad-Elazari H Reelick MF Bloem BR Giladi N. The role of the frontal lobe in complex walking among patients with Parkinson's disease and healthy older adults: an fNIRS study. Neurorehabil Neural Repair. (2016) 30:963–71. doi: 10.1177/1545968316650426 27221042

[19] Wang L Li F Tang L. Chronic effects of different exercise types on brain activity in healthy older adults and those with Parkinson's disease: A systematic review. Front Physiol. (2022) 13:1031803. doi: 10.3389/fphys.2022.1031803 36518109 PMC9742540

[20] Särkämö T Tervaniemi M Laitinen S Numminen A Kurki M Johnson JK. Cognitive, emotional, and social benefits of regular musical activities in early dementia: randomized controlled study. Gerontologist. (2014) 54:634–50. doi: 10.1093/geront/gnt100 24009169

[21] Hou X Zhang Z Zhao C Duan L Gong Y Li Z. NIRS-KIT: a MATLAB toolbox for both resting-state and task fNIRS data analysis. Neurophotonics. (2021) 8:010802. doi: 10.1117/1.NPh.8.1.010802 33506071 PMC7829673

[22] Lin JP Feng HS Zhai H Shen X. Cerebral hemodynamic responses to the difficulty level of ambulatory tasks in patients with Parkinson's disease: A systematic review and meta-analysis. Neurorehabil Neural Repair. (2021) 35:755–68. doi: 10.1177/15459683211028548 34171982

[23] Zou L Sasaki JE Wei GX Huang T Yeung AS Neto OB. Effects of mind⁻Body exercises (Tai chi/yoga) on heart rate variability parameters and perceived stress: A systematic review with meta-analysis of randomized controlled trials. J Clin Med. (2018) 7(11):404. doi: 10.3390/jcm7110404 PMC6262541 30384420

[24] Sungkarat S Boripuntakul S Kumfu S Lord SR Chattipakorn N. Tai chi improves cognition and plasma BDNF in older adults with mild cognitive impairment: A randomized controlled trial. Neurorehabil Neural Repair. (2018) 32:142–9. doi: 10.1177/1545968317753682 29353543

[25] Petzinger GM Fisher BE McEwen S Beeler JA Walsh JP Jakowec MW. Exercise-enhanced neuroplasticity targeting motor and cognitive circuitry in Parkinson's disease. Lancet Neurol. (2013) 12:716–26. doi: 10.1016/S1474-4422(13)70123-6 PMC3690528 23769598

[26] Tao J Chen X Egorova N Liu J Xue X Wang Q. Tai Chi Chuan and Baduanjin practice modulates functional connectivity of the cognitive control network in older adults. Sci Rep. (2017) 7:41581. doi: 10.1038/srep41581 28169310 PMC5294576

[27] Wei GX Dong HM Yang Z Luo J Zuo XN. Tai Chi Chuan optimizes the functional organization of the intrinsic human brain architecture in older adults. Front Aging Neurosci. (2014) 6:74. doi: 10.3389/fnagi.2014.00074 24860494 PMC4029006

[28] Koelsch S. Brain correlates of music-evoked emotions. Nat Rev Neurosci. (2014) 15:170–80. doi: 10.1038/nrn3666 24552785

[29] Di Biasio F Vanacore N Fasano A Modugno N Gandolfi B Lena F. Neuropsychology, neuroimaging or motor phenotype in diagnosis of Parkinson's disease-dementia: which matters most? J Neural Transm (Vienna). (2012) 119:597–604. doi: 10.1007/s00702-011-0733-3 22160550

[30] Wayne PM Walsh JN Taylor-Piliae RE Wells RE Papp KV Donovan NJ. Effect of tai chi on cognitive performance in older adults: systematic review and meta-analysis. J Am Geriatr Soc. (2014) 62:25–39. doi: 10.1111/jgs.12611 24383523 PMC4055508

[31] Elbek JA Nørgaard B Pedersen T Thuesen J. Non-pharmacological rehabilitation for people living with advanced Parkinson's disease: A scoping review of interventions. Parkinsonism Relat Disord. (2025) 133:107317. doi: 10.1016/j.parkreldis.2025.107317 39922750

